# The New Scheme of the Hospital Officers' Association

**Published:** 1920-12-25

**Authors:** 


### HOSPITAL SOCIAL SERVICE.

### The New Scheme of the Hospital Officers' Association.

It will be remembered that we set forth in these pages (November 6, p. 131) a full account of the work of that excellent body, the Almoner's Council, which has provided the hospital work with so many useful officers on the side dealing with social service. Recently the Council has made arrangements whereby students selected for training may obtain a considerable part of the qualifying training.

But a training which occupies a period of two years, in which the student has to keep herself and pay fairly substantial fees, has disadvantages as well as advantages. It excludes to too far an extent the actual worker in hospitals, who, while having to earn her living, is still ambitious of being promoted to a higher office. For such a one the recent extension of the Educational Section of the Incorporated Association of Hospital Officers offers an opportunity. In this scheme we gather that it is not intended to compete with the Almoner's Council. On the face of it, the course of training, although always open to improvement, is not on the same high level. But undoubtedly there is a field of occupation, possibly in the smaller hospitals, for a less highly trained officer, especially for the assessment of patients under the rapidly extending system of patients' payments as well as for the purely social work, which is the first consideration in the functions of an almoner. Moreover, the supply of trained social workers at present does not meet the demand.

The word "almoner" itself presents some difficulty to one wishing to understand the office and not possessing an inner knowledge of hospital routine. The title Lady Almoner is generally acknowledged to be pretentious, but dropping a qualification which now appears to belong mostly to char-ladies, we are left with a title which, in the circumstances, is singularly inexpressive. Moreover, there is sure to be some confusion between Council Almoners and Association Almoners, which either body would wish to avoid. If the name must be retained, and all alternatives to it suggested heretofore are very clumsy, we would suggest that the officers under the newer scheme be called Incorporated Almoners, as incorporation under a legal form is a valued possession of the Hospital Officers' Association, and must give a certain sense of security to the holder of a diploma.

We print a summary of the Association's scheme:

Course of Instruction for Almoners, Inquiry Officers, and Social Workers, &c.

Candidates must not be less than twenty years of age on January 1, 1921, and must have served immediately before that date for at least six months in the office of an almoner, inquiry officer, or out-patient officer of a voluntary hospital in Great Britain or Ireland, to be approved by the Educational Committee of the Hospital Officers' Association, whose decision in this matter shall be final.

Candidates must be certified as having a working knowledge of typewriting and shorthand.

Part I.—January-March, 1921.

Elementary lectures of the General Educational Course: Group 1. Committee Work and General Administration. Group 2. Financial and Statistical. Group 4. Historical and Theoretical. For full particulars see General Syllabus.

Part II.—April-July, 1921.

Selected course of lectures at King's College for Women, and London School of Economics (University of London), on the Social Side of organisations and the Social Work of the Present Day.

A certificate will be required that attendance at not less than 75 per cent. of the lectures has been made.

Fuller particulars will be announced later.

Part III.—October-December, 1921.

Attendance at lectures of the Educational Section of the H.O.A. Subjects: Public, Private, and Mutual Assistance of the Poorer Classes of England; Work in the Almoner's Office of a Voluntary Hospital; Collection of Fees from Patients, and Book-keeping in respect thereto.

The lectures to be taken will be indicated later in the General Syllabus.

Examinations will be held at the end of each part, and, subject to the result of these, a certificate will be issued by the Incorporated Association of Hospital Officers qualifying for a position of almoner or inquiry officer. Applications to spread the course over a longer period may be made, and will be considered on their merits by the Educational Committee. Candidates must hold throughout the course full-time occupation in a voluntary hospital on the side dealing with the reception, registration or examination of patients.

*Fees.*—The fees for each course of the Incorporated Association of Hospital Officers' lectures will be 5s. for members of the Association, and 10s. for non-members of the Association. It is expected that the cost of the lectures under the University of London will not exceed 30s. for the course.

All inquiries in connection with this course should be made to the Hon. Secretary, Mr. H. F. Pitt, City of London Hospital for Diseases of the Chest, Victoria Park, E. 2.

